# Inhibition of NAMPT aggravates high fat diet-induced hepatic steatosis in mice through regulating Sirt1/AMPKα/SREBP1 signaling pathway

**DOI:** 10.1186/s12944-017-0464-z

**Published:** 2017-04-27

**Authors:** Ling-Fang Wang, Xiao-Nv Wang, Cong-Cong Huang, Long Hu, Yun-Fei Xiao, Xiao-Hui Guan, Yi-Song Qian, Ke-Yu Deng, Hong-Bo Xin

**Affiliations:** 1 Institute of Translational Medicine, Nanchang University, 999 Xuefu Load, Honggutan District, Nanchang, 330031 China; 2 School of Life Sciences, Nanchang University, Nanchang, 330031 China

**Keywords:** Nampt, Nad^+^, Nafld, FK866, Sirt1, AMPKα, Mouse

## Abstract

**Background:**

Nonalcoholic fatty liver disease is one of the most common liver diseases in the world and is a typical hepatic manifestation of metabolic syndrome which is characterized with lipid accumulation in liver. Nicotinamide phosphoribosyltransferase (NAMPT) has been recently identified as an enzyme involved in nicotinamide adenine dinucleotide (NAD^+^) biosynthesis and plays an important role in cellular metabolism in variety of organs in mammals. The aim of this study was to investigate the effects of NAMPT on high fat diet-induced hepatic steatosis.

**Methods:**

Hepatic steatosis model was induced by high fat diet (HFD) in C57BL/6 mice in vivo. HepG2 and Hep1-6 hepatocytes were transfected with NAMPT vector plasmid or treated with NAMPT inhibitor FK866 and then incubated with oleic acid. Lipids accumulation was examined by HE staining or oil red staining. Quantitative RT-PCR and Western blot were used to measure expressions of the genes involved in lipogenic synthesis.

**Results:**

FK866 significantly promoted liver steatosis in the mice fed with HFD and hepatic lipid accumulation in vitro, accompanied by the increases of the expressions of lipogenic genes such as sterol regulatory element-binding protein 1 (SREBP1) and fatty acid synthase (FASN). Nicotinamide mononucleotide (NMN) and NAD^+^ significantly rescued the actions of FK866 in vitro. In contrast, overexpression of NAMPT in HepG2 and Hep1-6 hepatocytes ameliorated hepatic lipid accumulation. In addition, FK866 decreased the protein levels of Sirt1 and phospho-AMPKα in liver of the HFD fed mice. Furthermore, Resveratrol, a Sirt1 activator, significantly reduced lipogenic gene expressions, while EX-527, a Sirt1 specific inhibitor, had the opposite effects.

**Conclusion:**

Our results demonstrated that inhibition of NAMPT aggravated the HFD- or oleic acid-induced hepatic steatosis through suppressing Sirt1-mediated signaling pathway. On the one hand, the inhibition of NAMPT reduced the production of NAD^+^ through inhibiting the NAD^+^ salvage pathway, resulting in the decrease of Sirt1 activity, and then attenuated the deacetylation of SREBP1 in which the inhibition of SREBP1 activity promoted the expressions of FASN and ACC. On the other hand, the reduced Sirt1 activity alleviated the activation of AMPKα to further enhance SREBP1 activities.

**Electronic supplementary material:**

The online version of this article (doi:10.1186/s12944-017-0464-z) contains supplementary material, which is available to authorized users.

## Background

Over the past decade, the prevalence of nonalcoholic fatty liver disease (NAFLD) is increasing globally, and it has become the predominant cause of chronic liver disease in the world [1]. The morbidity of NAFLD varies between 20% and 50% in the western countries [2] and NAFLD is associated with many diseases such as obesity, type 2 diabetes and hepatocellular carcinoma [3–5]. Although abnormal liver lipid accumulation is considered to be one of the main causes of NAFLD, the molecular mechanisms of NAFLD are not fully elucidated.

Hepatic lipid accumulation results from an imbalance between lipid deposition and removal, which is associated with increased hepatic lipogenesis, augmented lipid uptake and/or decreased triglyceride export or β-oxidation [6, 7]. Hepatic lipid synthesis is regulated by many important transcription factors such as liver X receptor (LXR), carbohydrate response element binding protein (ChREBP) and sterol regulatory element–binding protein 1C (SREBP1C) [8–10]. As a major transcription factor, SREBP1 has been reported to widely regulate the key enzymes of synthesizing fatty acids including fatty acid synthase (FAS), acetyl-CoA carboxylase (ACC) and stearoyl-CoA desaturase (SCD1) [9, 11, 12]. Moreover, it has been found that the phosphorylation of AMPKα at its Ser372 suppressed the cleavage and nuclear translocation of SREBP-1c and then further repressed the expressions of the SREBP1C-mediated target genes in hepatocytes when the cells were treated with high glucose, leading to reduction of lipogenesis and lipid accumulation [13].

Nicotinamide phosphoribosyltransferase (NAMPT) is a highly conserved 52 kDa protein which is expressed in nearly all tissues/cells [14]. NAMPT has both intra- and extracellular forms in mammals. It is an important regulator of the intracellular nicotinamide adenine dinucleotide (NAD^+^) pool through regulating the rate-limiting step in the mammalian NAD^+^ salvage pathway from NAM [15]. The intracellular NAMPT (iNAMPT) has been proposed to have cell protective benefits via influencing the activity of NAD-dependent enzymes, such as Sirtuins due to its boosting NAD^+^ level [16]. The extracellular NAMPT (eNAMPT) in addition to its enzymatic function, it has cytokine-like activity. Although there are some debates, several reports suggest that circulating levels of eNampt may be closely related to obesity, NAFLD, atherosclerosis and diabetes mellitus [17–20]. However, it has been recently reported that iNampt was downregulated in NAFLD and had anti-apoptosis effects [21]. Moreover, other studies have found that aging-associated NAD^+^ deficiency was a critical risk factor for NAFLD, which resulted from NAMPT-controlled NAD^+^ salvage compromised in liver [22]. Although these results indicated that NAMPT could play a role in NAFLD, the effects of NAMPT on the pathogenesis of these disorders, especially in hepatic steatosis were largely unknown. Recently, we observed that inhibition of NAMPT significantly aggravated the high fat diet-induced obesity in mice (unpublished data). In the present study, the potential role of intracellular NAMPT in regulating hepatic lipid metabolism was explored. Our results showed that FK866, an NAMPT inhibitor, significantly promoted hepatic lipid accumulation in vitro and in vivo by promoting the transcriptional activity of SREBP1, which functions as an upstream regulator of FASN and ACC expressions. In addition, we observed that the effects could be rescued by NMN and NAD^+^, or overexpression of NAMPT. Furthermore, we demonstrated that the inhibition of NAMPT-mediated the production of intracellular NAD^+^ promoted hepatic lipid synthesis through inhibiting Sirt1 signaling pathway, in turn, activating AMP-activated protein kinase (AMPKα) and then inhibiting SREBP1 activity.

## Methods

### Reagents

FK866 (Cat.No: F8557), Resveratrol (Cat.No: R5010), EX527 (Cat.No: E7034), NAD^+^ (Cat.No: N5755), NMN (Cat.No: N3501), Oleic acid (O1383) and Oil Red O (Cat. No: O0625) were purchased from Sigma.

### Animals and diets

Male C57BL/6 mice with 8 week old age were housed in an animal room with a 12-h:12-h light/dark cycle under controlled environment (22 ± 3 °C, 50–60% relative humidity) and initially fed with standard diet chow for 1 week to adapt the housing condition. Thereafter, the animals were randomly divided into four groups (*n* = 5) as follows: 1) normal diet (ND), 2) ND + FK866, 3) High Fat diet (HFD), and 4) HFD + FK866. The mice were first fed either ND or HFD (60% kcal as fat, D12492; Research Diets Inc.) with or without FK866 (2 mg/kg/day, IP) for 1 week. Then, the mice were continually fed with ND or HFD for 12 weeks. All the experimental procedures were approved by Nanchang University Institutional Animal Research Committee and were curried out in accordance with Jiangxi Province Laboratory Animal Care Guidelines for the use of animals in research.

### Cell culture and treatment

HepG2 and Hep1-6 cells were cultured in 6-well plates in Dulbecco’s modified Eagle’s medium (DMEM) (Gibco, Grand Island, NY, USA) with 10% fetal bovine serum (FBS, Gibco) and 1% penicillin/streptomycin (Gibco) in a 5% humidified CO_2_ incubator at 37 °C. The cells were used for the experiments when they were in the exponential phase of growth. At about 80% confluence, the cells were treated with the fresh media containing NAMPT inhibitor FK866, or Sirt1 activator/inhibitor, respectively. After 24 h treatment, the cells were harvested for further experiments.

### Cell transfections

The Myc-DDK-tagged NAMPT plasmid was purchased from Origene (MR207867) and the cells were transfected with NAMPT vector plasmid and empty vector plasmid using lipo3000 transfection reagent (Invitrogen). Briely, 1.0 × 10^6^ cells were seeded in 6-well plates in DMEM. The plasmid DNA-lipid complexes were prepared according to the instructions and the complexes were incubated for 5 min at room temperature. The DNA-lipid complexes were added into the cells with 70%-80% of confluence, and incubated with the complex alone or plus oleic acid (0.5 mM) for 24 h.

### Oil red O staining

HepG2 and Hep1-6 cells were cultured in 24-well plates. Briefly, At 70–80% confluence, the cells were given the appropriate treatment for 24 h, then washed 3-4 times with PBS, and fixed with 4% paraformaldehyde for 1 h, lastly staining with Oil-Red O for 1 h, and then washed 3 times with PBS. Finally, the cells were examined by light microscopy (magnification, 40×). For quantitative analysis of lipid accumulation, isopropanol was added to the stained culture plate and the absorbance was recorded at 490 nm.

### Triglyceride measurement

The content of intracellular triglycerides in the HepG2 and Hep1-6 cells with inhibition or overexpression of NAMPT was measured using triglyceride assay kit (PPLYGEN, Beijing, China) according to the manufacturer’s instruction, and normalized to total protein concentrations.

### Protein extraction from liver tissues and cultured cells

Livers were isolated from eight-week-old mice which fed with ND or HFD for 12 weeks, and then washed with cold PBS followed by being lysed with RIPA buffer (0.5% NP-40, 0.1% sodium deoxycholate, 150 mM NaCl, 50 mM Tris-Cl, pH 7.5). After homogenization, the proteins were obtained by centrifugation at 4 °C for 5 min at 12000 rpm, then the supernatant was collected for further analysis and the pellet was discarded. And protein concentration was determined using the Bradford reagent (Bio-Rad) with BSA as a standard. The cultured cells were first washed twice with cold PBS, and then lysed with 2 × loading buffer (Bio Rad). Subsequently, scraped off the adherent cells, the lysates were collected for further analysis.

### Western blot analysis

Total proteins were separated by SDS-PAGE, and transferred to a polyvinylidene difluoride (PVDF) membrane and then the membranes were incubated overnight at 4 °C with various primary antibodies including anti-phospho-AMPKα (Thr172), anti-AMPKα, anti-ACC, anti-tubulin, anti-FASN, anti-SIRT1, anti-SREBP1 and anti-GAPDH, respectively. The membranes were incubated with appropriate horseradish peroxidase (HRP)-conjugated secondary antibody for 1 h at room temperature and visualized using enhanced chemiluminescence western blotting detection reagents.

### Total RNA extraction and real-time RT-PCR

Total RNA was prepared using Trizol reagent (Invitrogen) according to the manufacturer’s protocol. The RNA was reversely transcribed using the Takara high capacity cDNA synthesis kit. Synthesized cDNA was amplified by real-time RT**-**PCR (Vii7) using SYBR premix Ex TaqII (TaKaRa) and specific primers. The primer sequences were summarized in Table 1. The PCR was curried out with the condition of denaturation for 40 cycles at 95 °C for 30 s, annealing at 60 °C for 34 s, and extension at 60 °C for 1 min. Relative mRNA levels were calculated by the 2^-ΔΔCT^ method and normalized by GAPDH.

**Table 1:** QPCR primers used in this study

Gene Name	Forward	Reverse
hNAMPT	TTGCTGCCACCTTATC	AACCTCCACCAGAACC
hFASN	AAGGACCTGTCTAGGTTTGATGC	TGGCTTCATAGGTGACTTCCA
hSREBP1	CGCAAGGCCATCGACTACAT	GACTTAGGTTCTCCTGCTTGAGTTTC
hACC	TCGCTTTGGGGGAAATAAAGTG	ACCACCTACGGATAGACCGC
hSCD1	GCAGGACGATATCTCTAGCT	GTCTCCAACTTATCTCCTCCATTC
hGAPDH	CAGGGCTGCTTTTAACTCTGGT	GATTTTGGAGGGATCTCGCT
mNAMPT	TCGGTTCTGGTGGCGCTTTGCTAC	AAGTTCCCCGCTGGTGTCCTATGT
mFASN	GGAGGTTGCTTGGAAGAG	CTGGATGTGATCGAATGCT
mSREBP1	AGGTGTATTTGCTGGCTTGGT	AGAGATGACTAGGGAACTGTGTGT
mACC	GGACCACTGCATGGAATGTTAA	TGAGTGACTGCCGAAACATCTC
mSCD1	GCTGGGCAGGAACTAGTGAG	GAAGGCATGGAAGGTTCAAA
mGAPDH	AGCCAAAAGGGTCATCATCT	GGGGCCATCCACAGTCTTCT

### Histopathological examination

Ethanol-dehydrated, xylene-treated, and paraffin-embedded tissue sections were sliced at 5 μm. All liver sections were stained with haematoxylin and eosin (H&E) according to standard protocol. The degree of hepatic steatosis was determined with light microscopy.

### Statistical analysis

Data and results were reported as means ± SE. Statistical comparisons were performed with Student’s t test. Statistical significance was set at *p < 0.05, **p < 0.01, ***p < 0.001.

## Results

### Inhibition of NAMPT aggravates hepatic lipid accumulation in vivo

**Fig. 1:**
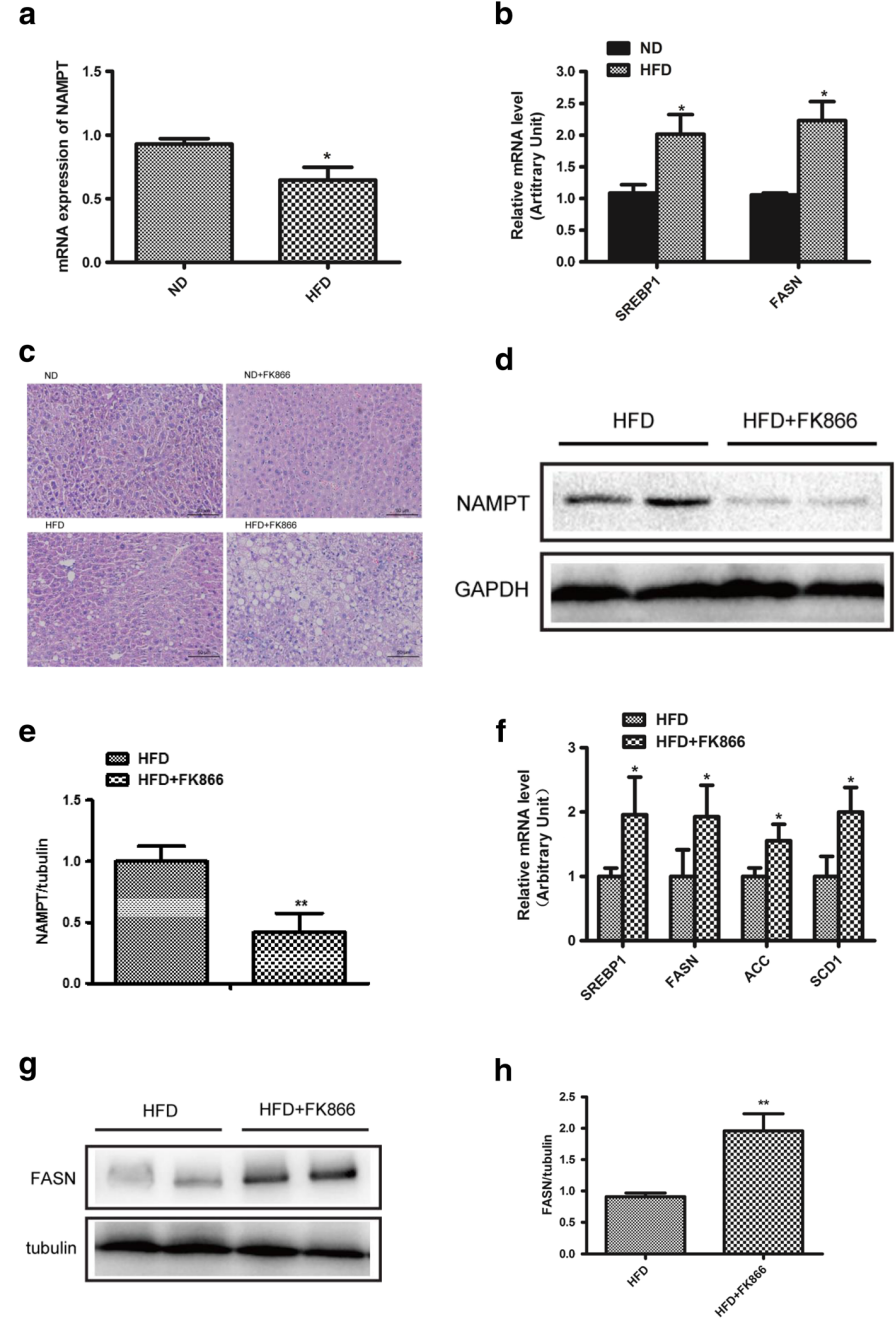
Inhibition of NAMPT aggravates hepatic lipid accumulation in vivo. FK866 (NAMPT inhibitor, 2 mg/kg/day, IP) was administrated into male C57BL/6 mice for 1 week with normal diet (ND) or high fat diet (HFD) and then, the mice were continually fed with ND or HFD for 12 weeks. **a** The relative expressions of NAMPT by real-time PCR analysis in liver of the mice. **b** Quantitative analysis of the expressions of the lipid synthetic genes by RT-PCR in liver from the mice. **c** The morphological analysis of Hematoxylin and eosin staining in liver from the mice. **d** The expression of NAMPT in liver from the HFD-fed mice with a NAMPT inhibitor FK866. **e** Quantitative analysis of NAMPT protein level from western blot bands. **f** Quantitative analysis of the expressions of the lipid synthetic genes by RT-PCR in liver from the mice. **g** The images of FASN protein by Western blot analysis in liver from the mice. **h** Quantitative analysis of FASN protein level from western blot bands. The data represent the mean ± SEM. *N* = 5, *P < 0.05 and **P < 0.01 versus the control

To determine the potential role of NAMPT in lipid metabolism, we first examined NAMPT expressions in the liver from normal diet (ND)- and high fat diet (HFD)-fed mice. The results showed that NAMPT expression was markedly decreased in liver tissue from the mice fed with HFD compared with the mice fed with normal diet (Fig. 1a). Moreover, the expressions of lipogenic genes including SREBP1 and FASN were significantly increased in the livers of HFD-fed mice compared with the mice fed with normal diet (Fig. 1b). To clarify whether NAMPT affects hepatic lipid accumulation, we examined the lipogenesis in the HFD-fed mice with a NAMPT inhibitor (FK866). The C57BL/6 mice were administrated with FK866 (2 mg/kg/day, ip) or vehicle for 1 week and then the mice were fed with HFD for additional 12 weeks. The H&E staining showed that there was a significant increase of hepatic lipid deposition in the mice fed with HFD compared with control mice, and FK866 significantly promoted the lipid accumulation in mice treated with HFD compared with the mice only fed with HFD (Fig. 1c). In addition, we found that NAMPT protein level was significantly decreased in FK866-treated mice under HFD (Fig. 1d-e). Real-time PCR analysis indicated that the expressions of the lipogenic genes such as SREBP1, FASN, ACC and SCD1 were increased in the HFD-fed mice treated with FK866 (Fig. 1f). The expressions of the fatty acid oxidation genes including CPT1, ﻿MCAD and PPARα were not changed in FK866-treated mice compared with control mice (﻿Additional file 1: Figure S1). Consistent with the alternations of mRNA, the protein levels of FASN were also increased in the liver of FK866 treated mice (Fig. 1g-h). These results indicated that FK866-induced inhibition of NAMPT significantly aggravated hepatic lipid accumulation in HFD-fed mice through enhancing lipogenesis.

### Inhibition of NAMPT promotes lipid accumulation in HepG2 cells

**Fig. 2:**
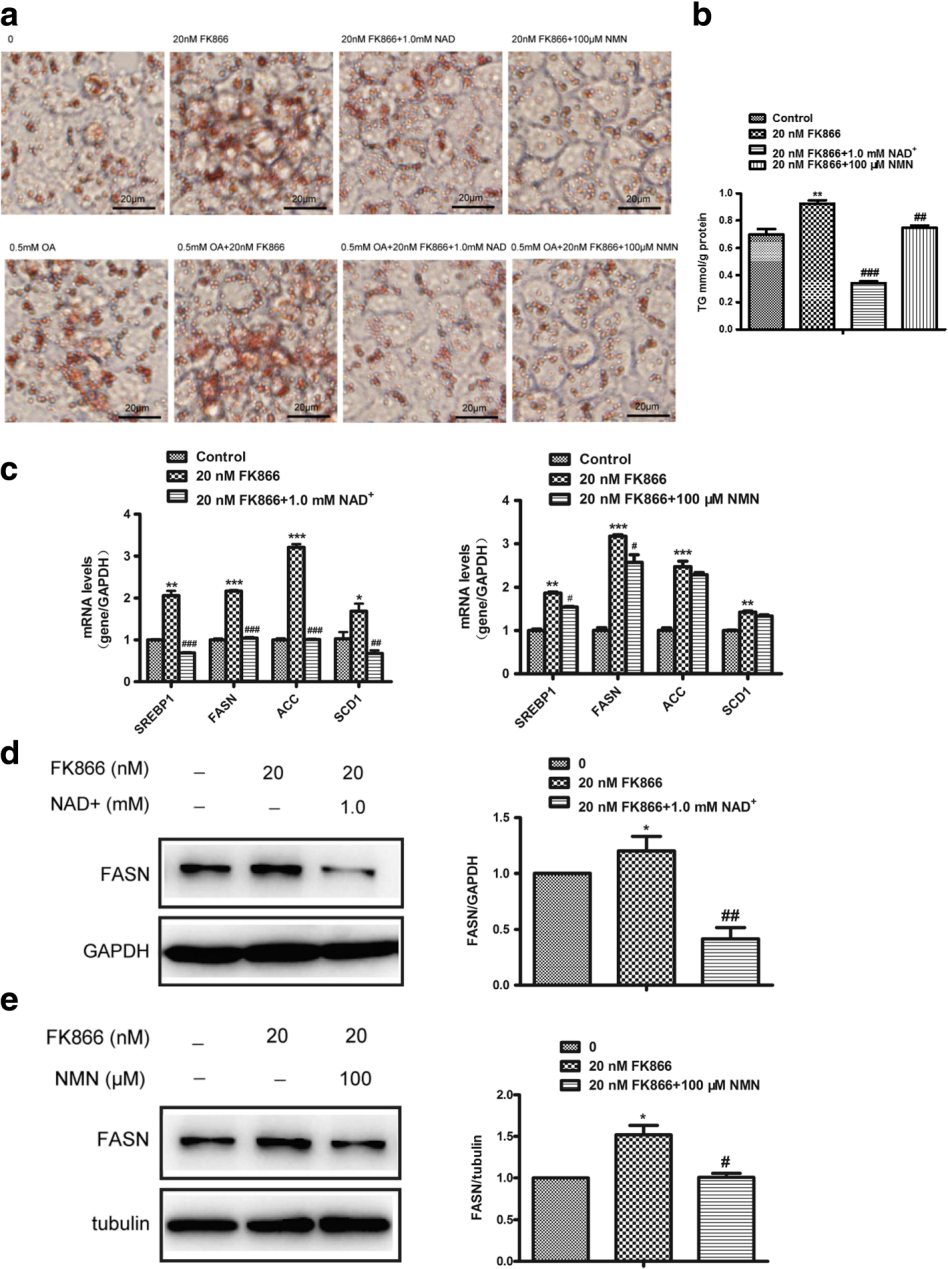
Inhibition of NAMPT aggravates hepatic lipid accumulation in vitro. The hepatic steatosis was induced by oleic acid (OA, 0.5 mM) in HepG2 cells and the effects of FK866 (20 nM) on lipogenesis in hepatic cells were detected with or without NAD^+^ (1 mM) or NMN (100 μM) treatment for 24 h. **a** The images of Oil red O staining of HepG2 cells. **b** The intracellular TG level in HepG2 treated with FK866 or combined with NAD^+^ or NMN. **c** Quantitative analysis of the expressions of lipid synthetic genes by RT-PCR in HepG2 cells. **d** Western blots images (left) and quantitative analysis (right) of FASN expression in HepG2 cells treated with NAD^+^. **e** Western blots images (left) and quantitative analysis (right) of FASN expression in HepG2 cells treated with NMN. All results were analyzed based on three independent experiments. The data represent the mean ± SEM. *P < 0.05 and **P < 0.01 versus the control; ^#^ *P <* 0.05, ^# #^ *P <* 0.01 and ^# # #^ *P <* 0.001 versus FK866

In order to verify the results of experiment in vivo, we employed the HepG2 cell lines for further confirming the findings in vitro. The HepG2 cells were treated with NAMPT inhibitor FK866 (20 nM) with or without oleic acid (0.5 mM) for 24 h. Oil Red O staining revealed that there was a significant increase of the lipid accumulation after treated with FK866 (Fig. 2a). Moreover, the intracellular TG level was increased in cells treated with FK866, while the effects of FK866 could be rescued by exogenous NAD^+^ and NMN (Fig. 2b). Our results also showed that FK866 markedly increased the expressions of the lipogenic genes including SREBP1, FASN, ACC and SCD1 (Fig. 2c) and NAD^+^, but not nicotinamide mononucleotide (NMN), could completely reverse the effects of FK866 (Fig. 2a, c). Moreover, we found the expression of SREBP2 was consistent with SREBP1 in HepG2 cells treated with FK866 and combined with NAD^+^ or NMN (Additional file 1: Figure S2A). In addition, the changes of the FASN protein levels were consistent with the alternations of the mRNA expressions of the hepatic lipogenic genes in HepG2 cells treated with FK866 (20 nM) combined with or without NAD^+^ and NMN (Fig. 2d-e). Taken together, our results demonstrated that FK866-mediated inhibition of NAMPT aggravated hepatic lipid accumulation in vitro.

### Overexpression of NAMPT reverses oleic acid-induced lipid accumulation in hepatocytes

**Fig. 3:**
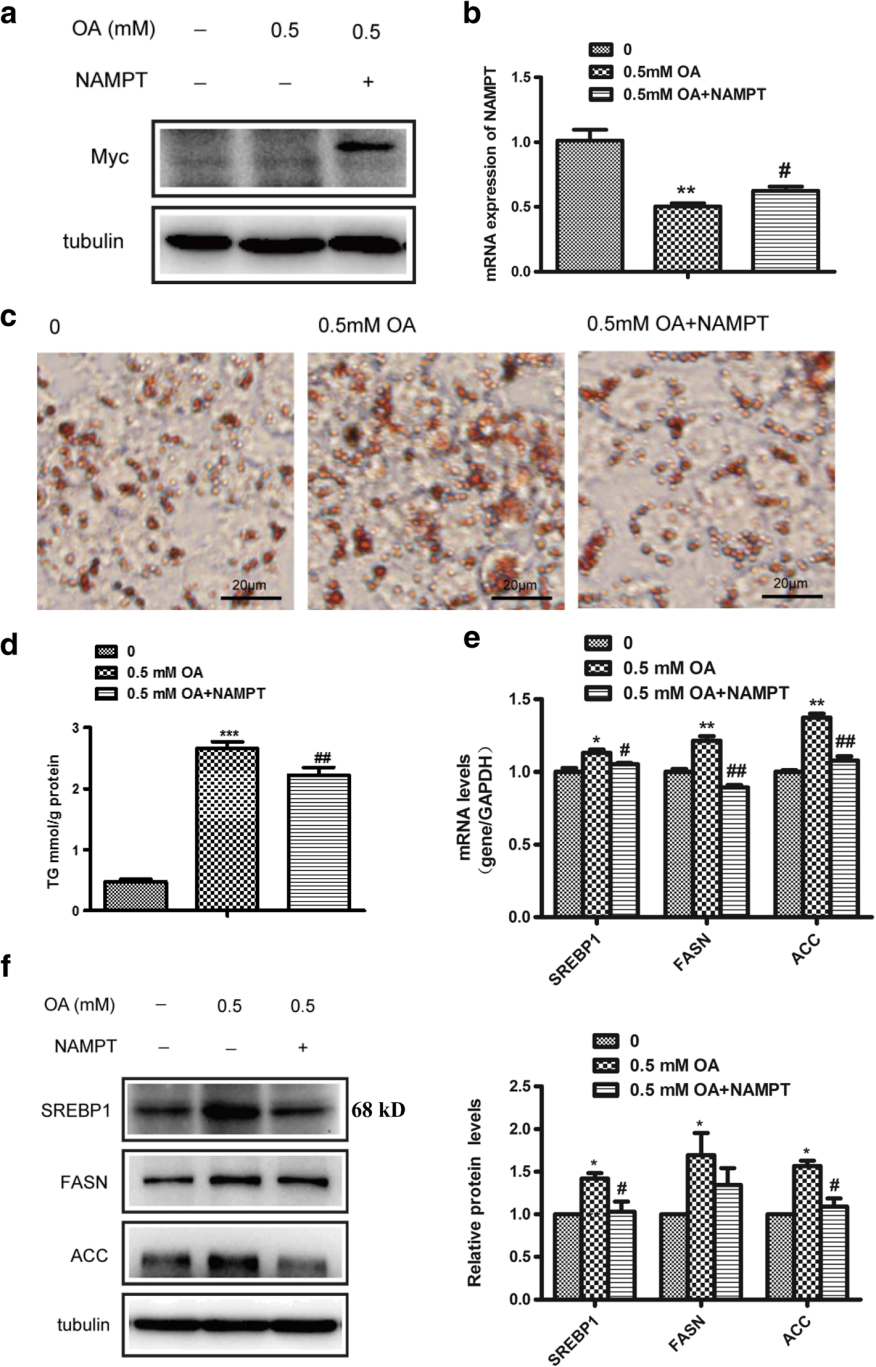
Overexpression of NAMPT reverses oleic acid-induced lipid accumulation in HepG2. The cells were transfected with NAMPT plasmid for 24 h and then treated with oleic acid (OA, 0.5 mM) for 24 h. **a-b** The images (**a**) and quantitative analysis (**b**) of NAMPT overexpression in HepG2 cells. **c** The images of Oil red O staining of HepG2 cells. **d** The intracellular TG level in HepG2 transfected with NAMPT plasmid or vector under OA stimulation. **e** Quantitative analysis of the mRNA expressions of lipid synthetic genes including SREBP1, FASN and ACC in HepG2 cells. **f** The western blot images (*left*) and quantitative analysis (*right*) of the protein levels of the lipid synthetic genes including SREBP1, FASN and ACC in HepG2 cells. All results were analyzed based on three independent experiments. The data represent the mean ± SEM. *P < 0.05 and **P < 0.01 versus the control; ^#^ *P <* 0.05 and^# #^ *P <* 0.01 versus OA

**Fig. 4:**
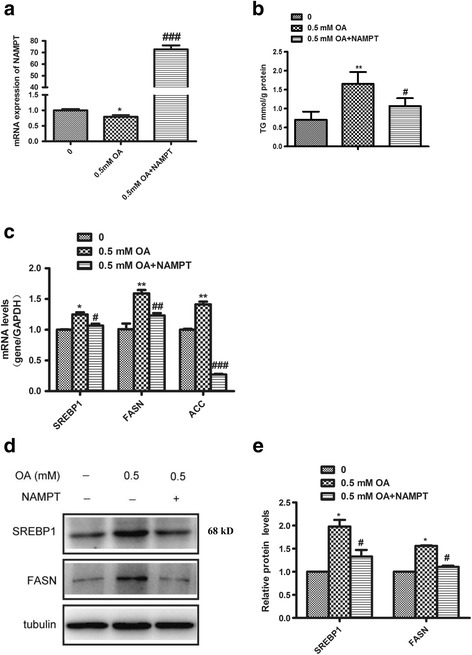
Overexpression of NAMPT reverses oleic acid-induced lipid accumulation in Hep1-6. The Hep1-6 cells were transfected with NAMPT plasmid for 24 h and then treated with oleic acid (0.5 mMt) for 24 h. **a** Quantitative analysis of overexpression of NAMPT by RT-PCR in Hep1-6 cells. **b** The intracellular TG level in Hep1-6 transfected with NAMPT plasmid or vector under OA stimulation. **c** Quantitative analysis of the mRNA expressions of lipid synthetic genes including SREBP1, FASN and ACC in Hep1-6 cells. **d** Western blots images of the expressions of SREBP1 and FASN in Hep1-6 cells. **e** Quantitative analysis of the expressions of lipid synthetic genes including SREBP1 and FASN by RT-PCR in Hep1-6 cells. All results were analyzed based on three independent experiments. The data represent the mean ± SEM. *P < 0.05 and **P < 0.01 versus the control; ^#^ *P <* 0.05,^# #^ *P <* 0.01 and ^## #^ *P <* 0.001 versus OA

To further confirm the role of NAMPT in lipid accumulation, HepG2 and murine liver cell lines Hep1-6 cells were transfected with NAMPT expression plasmid for 24 h and then treated with oleic acid (0.5 mM) for 24 h. As shown in Fig. 3a-b and Fig. 4a, both HepG2 and Hep1-6 cells transfected with NAMPT plasmid resulted in a significant increase of NAMPT mRNA and protein levels compared with control group, especially in Hep1-6 cells. In addition, we observed that the expressions of NAMPT were significantly reduced in HepG2 and Hep1-6 cells treated with oleic acid compared with vehicle (Figs. 3b and 4a). Oil Red O staining revealed a significant decrease in hepatic lipid deposition in HepG2 cells transfected with NAMPT (Fig. 3c). And we also found that overexpression of NAMPT significantly decreased intracellular TG level in both HepG2 and Hep1-6 cells induced by oleic acid (Figs. 3d and 4b). Importantly, the expressions of lipogenic genes such as SREBP1, FASN and ACC were suppressed in both HepG2 and Hep1-6 cells transfected with NAMPT before treated with oleic acid (Figs. 3e and 4c).﻿ We found SREBP2 mRNA level was also decreased in Hep1-6 cells overexpression of NAMPT (Additional file 1: Figure S2B). The similar results were observed for the expressions of SREBP1, FASN and ACC proteins in both type cells (Figs. 3f and 4d-e). These results indicated that overexpression of NAMPT ameliorated hepatic lipid accumulation in vitro.

### NAMPT inhibition-mediated lipid accumulation was associated with inhibition of Sirt1/SREBP1 signaling pathway

**Fig. 5:**
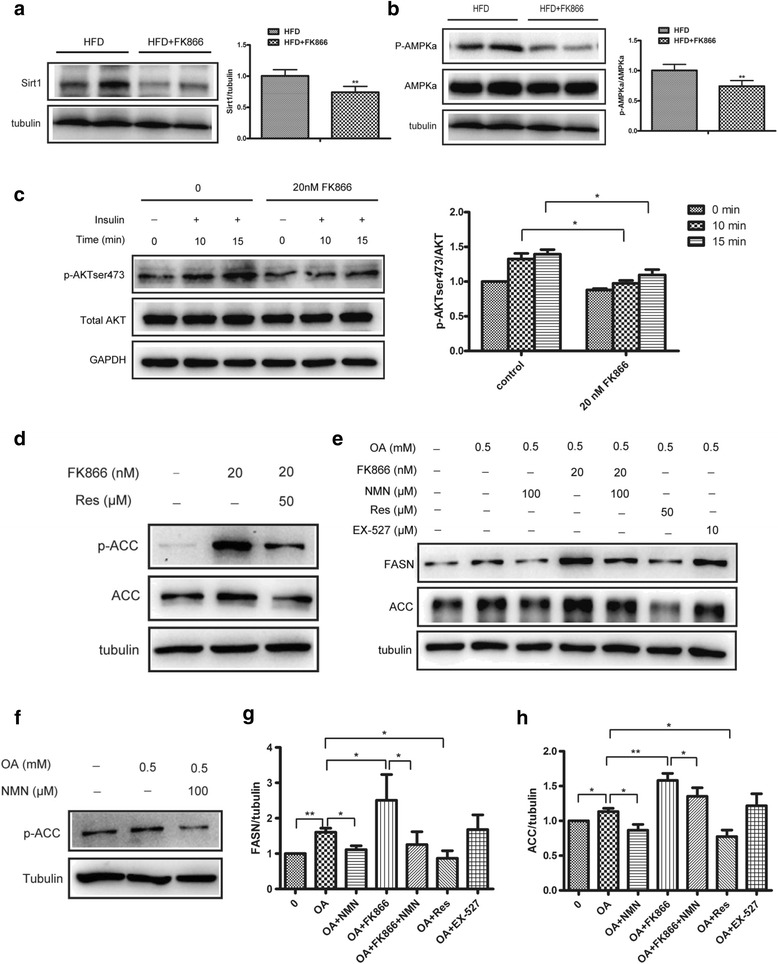
NAMPT inhibits lipid accumulation by targeting Sirt1/SREBP1 signaling. The WT and FK866-treated mice were fed with HFD for 12 weeks and the livers were isolated from the mice and the protein levels were determined by western blot analysis or the HepG2 cells were treated with different conditions. **a** Western blot image (*left*) and quantitative analysis (right) of the Sirt1 protein expression in liver. **b** Western blot images (*left*) and quantitative analysis (right) of the protein expressions of AMPKα and phosphorylated AMPKα at threonine 172 (p-AMPKα) in liver. **c** Western blot images (*left*) and quantitative analysis (*right*) of total AKT and phosphorylated AKT pathway in HepG2 cells with FK866 (20 nM) under insulin stimulation at different times. **d** Western blots images of ACC protein expression and phosphorylation in HepG2 cells treated by FK866 (20 nM) combined with Sirt1 activator (Resveratrol, 50 μM). **e-h** Western blot images (**e**-**f**) and quantitative analysis of FASN (**g**) and ACC (**h**) expressions in HepG2 cells treated by oleic acid (OA, 0.5 mM) combined with FK866 (20 nM) or NMN (100 μM) or Resveratrol (50 μM) or EX527 (10 μM). All results were analyzed based on three independent experiments. The data represent the mean ± SEM. *P < 0.05 and **P < 0.01 versus the control; ^#^ *P <* 0.05 and ^# #^ *P <* 0.01 versus OA

**Fig. 6:**
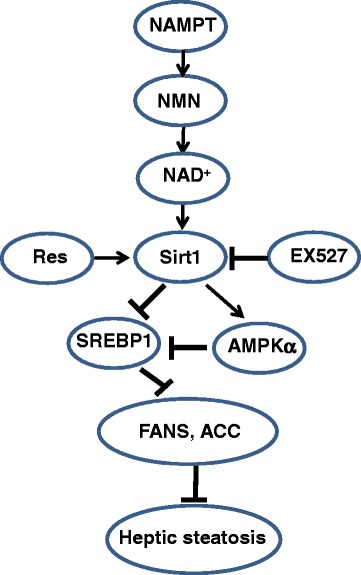
Mechanisms of NAMPT protecting liver from high fat diet-induced hepatic steatosis. NAMPT promotes the production of NAD^+^ by activating the NAD^+^ salvage pathway and in turn, the increased NAD^+^ as a substrate activates Sirt1 signaling pathways, alleviating high fat diet-induced hepatic steatosis in mice. On the one hand, the activation of Sirt1 promotes the deacetylation of SREBP1 in which the inhibition of SREBP1 activity finally results in inhibitions of the expressions of lipogenesis genes including FASN and ACC. On the other hand, Sirt1 directly activates AMPKα which further inhibits SREBP1 activities

It has been reported that NAMPT was a key rate-limiting enzyme in systemic NAD^+^ biosynthesis, and Sirt1, a NAD^+^-dependent deacetylase, played important roles in metabolic regulation. To elucidate the mechanisms of NAMPT-mediated inhibition of hepatic lipid accumulation, we first detected the expressions of Sirt1 in liver. Western blot analysis showed that the expression of Sirt1 was decreased in the FK866 treated mice compared to that of the control mice fed with HFD for 12 weeks (Fig. 5a). In addition, we examined the activation of AMPK which phosphorylated SREBP1 and ACC, and then switched metabolism from lipogenesis to lipid oxidation. Our results showed that FK866 led to a significant reduction in level of phosphorylated AMPKα in vivo (Fig. 5b). Many literatures demonstrated that abnormal hepatic lipid accumulation was always accompanied by hepatic insulin resistance. Based on the observations, we examined the effects of FK866 on insulin signaling in HepG2 cells and found that FK866 impaired insulin-stimulated Akt phosphorylation (Fig. 5c). Furthermore, to confirm the involvement of the Sirt1 pathway in the anti-lipogenic effects of NAMPT, HepG2 cells were treated with a Sirt1 activator (Resveratrol) or specific inhibitor (EX527). The FK866 combined with Resveratrol significantly reduced lipogenic gene ACC expression and phosphorylation compared with normal group (Fig. 5d). As expected, the expression levels of FASN and ACC were decreased in cells treated with oleic acid (OA) and resveratrol compared with cells treated with OA alone, while EX527 displayed the opposite influences (Fig. 5e-h). And we also found that NMN could reverse the effects of FK866 that promoted OA-mediated lipid synthesis. Taken together, these results demonstrated that NAMPT inhibited hepatic lipogenesis probably by activating Sirt1 and in turn, activating AMPKα, and finally inhibiting SREBP1 activity. The working model of NAMPT-mediated protecting liver from high fat diet-induced hepatic steatosis was presented in Fig. 6.

## Discussion

NAFLD is well demonstrated as the most common cause of liver disorders including simple steatosis, nonalcoholic steatohepatitis, liver cirrhosis and hepatocellular carcinoma [23]. The most notable characteristic of NAFLD is the accumulation of triglycerides in liver. It has been reported that dysregulation of lipid synthesis is one of the main reasons for abnormal lipid accumulation in the liver [24]. It was reported that NAFLD not only existed in mammals, also happened in vertebrates. It has been found that genetic ablation of solute carrier family 7a3a leaded to hepatic steatosis in zebrafish during fasting [25]. Recently, we observed that FK866 significantly aggravated the high fat diet-induced obesity in mice, in which the NAMPT inhibition-induced obesity might be involved in suppression of Sirt1-SREBP1-FASN signaling pathway in adipose cells (unpublished data). In the present study, we found that the expression of NAMPT was decreased in the liver of the mice fed with HFD. Furthermore, the expression of NAMPT was also reduced in HepG2 cells treated with oleic acid, which was the end product of de novo fatty acid synthesis [26]. In our study, an oleic acid-induced hepatic steatosis model was used to evaluate the effects of NAMPT on NAFLD in vitro since the accumulation of oleic acid played an important role in the development of hepatic steatosis in human beings. Our results suggested that NAMPT played a critical role in hepatic steatosis model in vivo and in vitro.

To further confirm the role of NAMPT in hepatic lipid metabolism, we first examined the effects of FK866, a highly specific NAMPT inhibitor, on HFD-induced hepatic steatosis. We observed that FK866 increased hepatic lipid deposition in mice fed with HFD, but not normal diet, indicating that FK866 promoted the synthesis of lipid in context of high fat diet in vivo. Besides, we found that FK866 significantly increased the expressions of lipid synthesis genes in HepG2 cells, suggesting that inhibition of NAMPT enhanced hepatic lipid synthesis in vivo and in vitro. In addition, we observed that NMN, an enzymatic product of NAMPT, could partially reverse the effects of FK866 in HepG2 cells. Previous studies showed that NMN supplementation improved diet and age-induced diabetes [27] and other damage such as vascular dysfunction, oxidative stress [28] and cognitive impairment [29]. NAD^+^, served as substrate in various signaling conduction pathways, plays an important role in age and metabolism-associated diseases [30, 31]. Our results showed that NAD^+^ could completely reverse the effects of FK866, suggesting that NAD^+^ played a central role in the development of NAFLD.

In order to further confirm the roles of NAMPT in NAFLD, the effects of overexpressing NAMPT on lipid accumulation have pursued in liver cell lines. Because the amino acid sequence of the human NAMPT has 96% homologous identity of mouse, the mouse NAMPT was used for gain-of-function experiments in both human HepG2 and mouse Hep1-6 cells. As expected, overexpression of NAMPT significantly attenuated the lipid accumulation and the expressions of lipogenic genes such as SREBP1 and its target genes including FASN and ACC. These results further demonstrated that NAMPT played an important role in lipid synthesis in liver.

SREBP1 is a critical regulator of lipid metabolism, in which it promotes expressions of lipogenic genes such as FASN and ACC, and it plays an important role in nonalcoholic fatty liver disease [32–34]. It has been reported that Sirt1, an NAD^+^-dependent protein deacetylase, can directly deacetylate SREBP1 [35]. Here, we found the expression of Sirt1 was reduced in FK866-treated mice fed with HFD. Furthermore, to further elucidate the mechanisms of Sirt1 in hepatic lipid metabolism, we examined the effects of EX527 (a specific inhibitor of Sirt1) and resveratrol (an activator of Sirt1) on lipogenesis in heptocytes and found that resveratrol significantly reduced lipogenic gene expressions, demonstrating that NAMPT-mediated inhibition of lipid metabolism was involved in NAD^+^-Sirt1 signaling pathway. Moreover, our results also showed that OA-induced expressions of FASN and ACC were remarkably downregulated by resveratrol and upregulated by EX527. These results indicated that NAMPT inhibited lipid synthesis through activating Sirt1 signaling pathway. Moreover, AMP-activated protein kinase (AMPK) is a major regulator of cellular energy homeostasis [36]. Recently, Li et al. reported that SREBP1c was one of the target proteins directly phosphorylated by AMPK [13]. In addition, Sirt1 was found to regulate AMPK activity via modulation of Lkb1, a major upstream kinase of AMPK in hepatic cells and animal models [37, 38], and in turn, AMPK enhanced Sirt1 activity by increasing cellular NAD^+^ levels [39]. Here, we provided in vivo evidence that the phosphorylation of AMPKα was significantly attenuated by FK866 in the mice fed with HFD. NAFLD is now considered to be the hepatic manifestation of the metabolic syndrome and has insulin resistance as its hallmark [40, 41]. In addition to the influence of NAMPT on lipid metabolism, we also found that FK866 markedly reduced Akt phosphorylation, indicating that NAMPT also improve insulin resistance in NAFLD through promoting insulin signaling in hepatocytes.

## Conclusion

The results from our present study demonstrated that NAMPT decreased hepatic lipid accumulation in vitro and in vivo, and the mechanisms of NAMPT-induced inhibition of hepatic lipid accumulation were mainly associated with its suppression of lipogenic genes including FASN and ACC expression through activating Sirt1 signaling pathway and then affecting the transcriptional activity of SREBP1 and activating AMPK. The findings of this study will broad our understanding of the roles of NAMPT in hepatic lipid metabolism and provide important insights in targeting NAMPT for treating liver steatosis and NAFLD.

## Additional file

Additional file 1: Figure S1. Quantitative analysis of the expressions of the fatty acid oxidation genes by RT-PCR in liver from the mice under HFD and treated with FK866. The values are expressed as the mean ± SE. *n* = 3. **Figure S2**. The expression of SREBP2 in HepG2 treated with FK866 or overexpression NAMPT. (A) Quantitative analysis of the mRNA expression of SREBP2 in HepG2 cells treated with FK866 or combined with NAD^+^ or NMN. (B) Quantitative analysis of the mRNA expression of SREBP2 in HepG2 cells transfected with NAMPT plasmid or vector under OA stimulation. All results were analyzed based on three independent experiments. The data represent the mean ± SEM. **P* < 0.05 and ***P* < 0.01 versus the control; #*P* < 0.05 and ## *P* < 0.01versus OA. (DOC 163 kb)

## Abbreviations

ACC: Acetyl-CoA carboxylase
AMPKα: AMP-activated protein kinase
FASN: Fatty acid synthase
HFD: High fat diet
NAD^+^: Nicotinamide adenine dinucleotide
NAFLD: Nonalcoholic fatty liver disease
NAMPT: Nicotinamide phosphoribosyltransferase
ND: Normal diet
NMN: Nicotinamide mononucleotide
OA: Oleic acid
SCD1: Stearoyl-CoA desaturase
SREBP1: Sterol regulatory element-binding protein 1
