# Supplementary material for: Inhibition of NAMPT aggravates high fat diet-induced hepatic steatosis in mice through regulating Sirt1/AMPKα/SREBP1 signaling pathway
Source: Lipids Health Dis. 2017 Apr 27;16:82. doi: 10.1186/s12944-017-0464-z (PMC5408374; doi:10.1186/s12944-017-0464-z)
Supplement: Additional file 1: Figure S1. — Quantitative analysis of the expressions of the fatty acid oxidation genes by RT-PCR in liver from the mice under HFD and treated with FK866. The values are expressed as the mean ± SE. n = 3. Figure S2. The expression of SREBP2 in HepG2 treated with FK866 or overexpression NAMPT. (A) Quantitative analysis of the mRNA expression of SREBP2 in HepG2 cells treated with FK866 or combined with NAD+ or NMN. (B) Quantitative analysis of the mRNA expression of SREBP2 in HepG2 cells transfected with NAMPT plasmid or vector under OA stimulation. All results were analyzed based on three independent experiments. The data represent the mean ± SEM. *P < 0.05 and **P < 0.01 versus the control; #P < 0.05 and ## P < 0.01versus OA. (DOC 163 kb) [file 12944_2017_464_MOESM1_ESM.doc]

**Supplementary Materials**

**
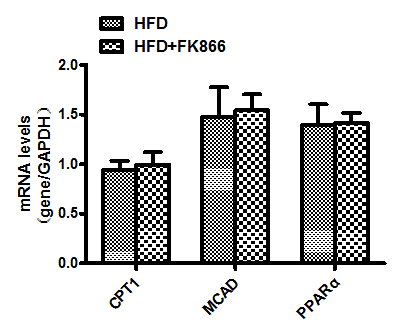
**

**Figure S1.** Quantitative analysis of the expressions of the fatty acid oxidation genes by RT-PCR in liver from the mice under HFD and treated with FK866. The values are expressed as the mean ± SE. *n* = 3.

**
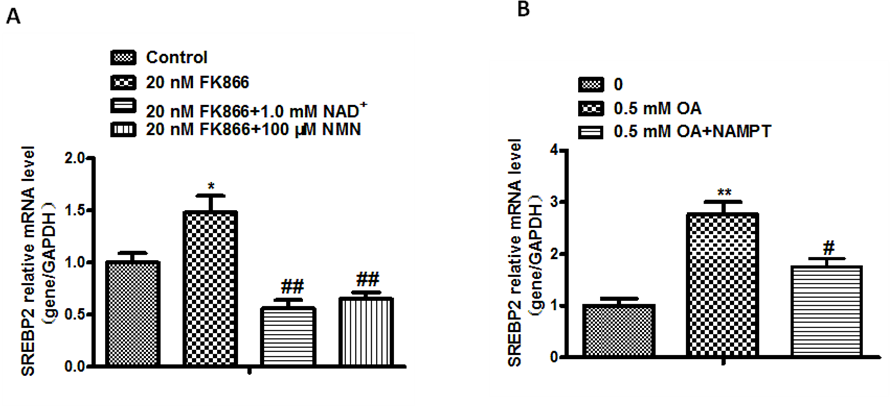
**

**Figure S2. The expression of SREBP2 in HepG2 treated with FK866 or overexpression NAMPT. (A)** Quantitative analysis of the mRNA expression of SREBP2 in HepG2 cells treated with FK866 or combined with NAD^+^ or NMN. **(B)** Quantitative analysis of the mRNA expression of SREBP2 in HepG2 cells transfected with NAMPT plasmid or vector under OA stimulation. All results were analyzed based on three independent experiments. The data represent the mean ± SEM. **P* <0.05 and ***P* <0.01 versus the control; #*P*<0.05 and ## *P* <0.01versus OA.
